# Correction: Neural, functional, and aesthetic impacts of spatially heterogeneous flicker: A potential role of natural flicker

**DOI:** 10.1371/journal.pone.0228810

**Published:** 2020-01-30

**Authors:** Melisa Menceloglu, Marcia Grabowecky, Satoru Suzuki

The images for Figs 3 and 4 are incorrectly switched. The image that appears as Fig 3 should be Fig 4, and the image that appears as Fig 4 should be Fig 3. The figure captions appear in the correct order.

**Fig 3:**
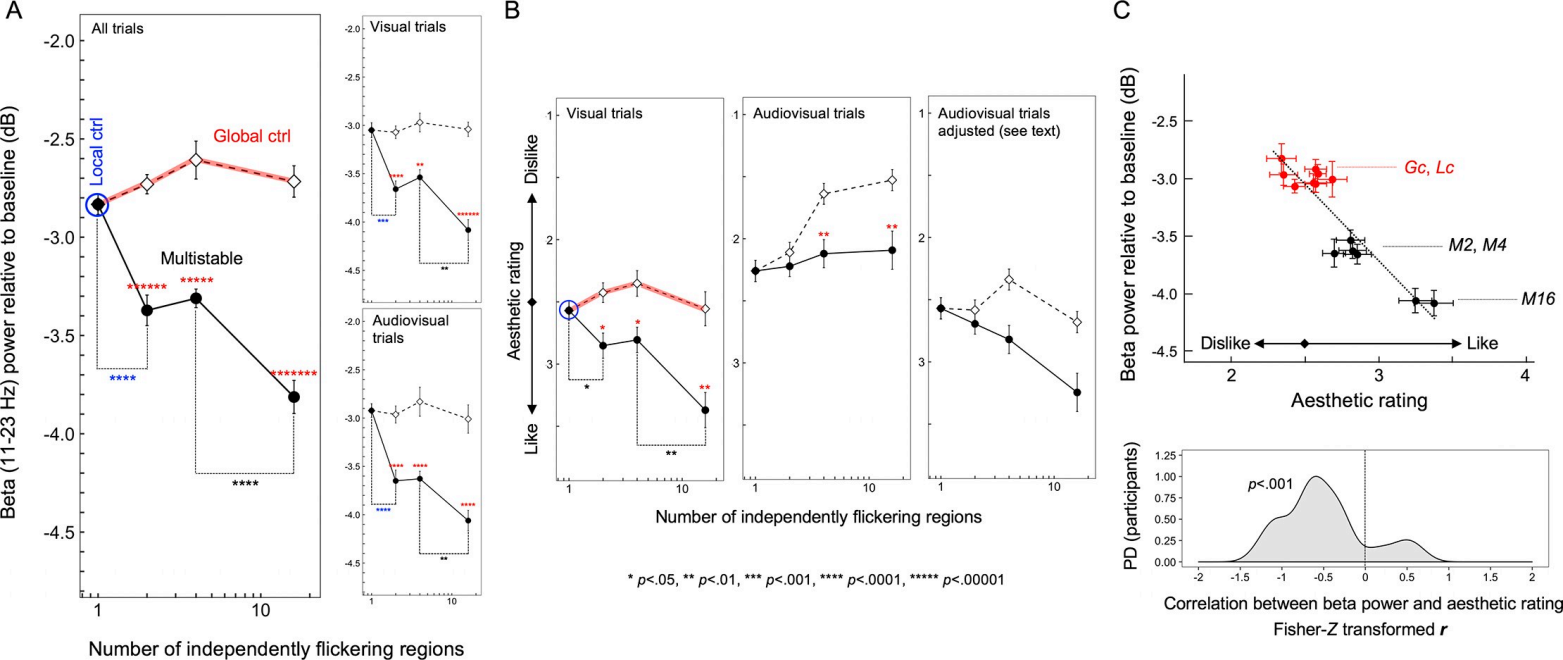
Beta-power reductions and aesthetic ratings from Experiment 1. (A) Time-averaged posterior EEG beta power (~11Hz to ~23Hz for which EEG power was significantly reduced for the multistable condition relative to all control conditions; see Fig 2) as a function of the number of independently flickering regions. Note that the local-control condition can be considered a “multistable” condition with the number of independently flickering regions being 1 because the rate of flicker was matched to the rate at which each region was independently flickered in the multistable condition. Overall, the multistable condition for all degrees of multistability (with 2, 4, or 16 independently flickering regions) significantly reduced beta power relative to the corresponding global-control conditions (red asterisks); the lowest degree of multistability (with only 2 independently flickering regions) significantly reduced beta power relative to the local-control condition (blue asterisks); the highest degree of multistability (with 16 independently flickering regions) significantly reduced beta power relative to the 4-region multistability (black asterisks). The pattern of results was similar when the data were examined separately for the visual and audiovisual trials (right panels). (B) Similar analyses on aesthetic ratings. When the y-axis is ordered from “strongly like” (4) to “strongly dislike” (1), the pattern of aesthetic ratings for the visual trials (left panel) strongly resembles the pattern of EEG beta-power reductions—the beta-power-reducing multistable conditions being generally preferred and the strongly beta-power-reducing 16-region multistability being especially preferred—suggesting that greater beta-power reductions are associated with higher aesthetic ratings. However, unlike the EEG beta-power reductions that were similar between the visual and audiovisual trials (see (A)), the aesthetic ratings were lowered in the audiovisual trials (middle panel). However, if we make the simple assumption that the synchronized click sounds (which sounded unpleasant to most participants) subtractively lowered aesthetic preferences without interacting with flicker effects, the appropriately adjusted ratings (right panel) resembles those from the visual trials (left panel) (see text for details). (C) Upper panel. Scatter plot showing the relationship between the posterior EEG beta-power reductions and aesthetic ratings (adjusted to exclude the general sound effects on ratings; see text for details) across the flicker conditions (*Lc*–local control; *Gc*–global control; *M2*, *M4*, and *M16*–multistable with 2, 4, and 16 independently flickering regions, respectively) with visual and audiovisual presentations. The negative relationship indicates that greater EEG beta-power reductions were associated with higher aesthetic ratings in response to different flicker conditions. Lower panel. Probability-density distribution (PD) of the Fisher-*Z* transformed *r* (Pearson’s correlation coefficient) for the above relationship computed for the individual participants; in support of the negative relationship above, the distribution of *r*_*z*_ is substantially shifted in the negative direction. The error bars represent ±1 *SEM* adjusted for repeated-measures comparisons (Morey, 2008).

**Fig 4:**
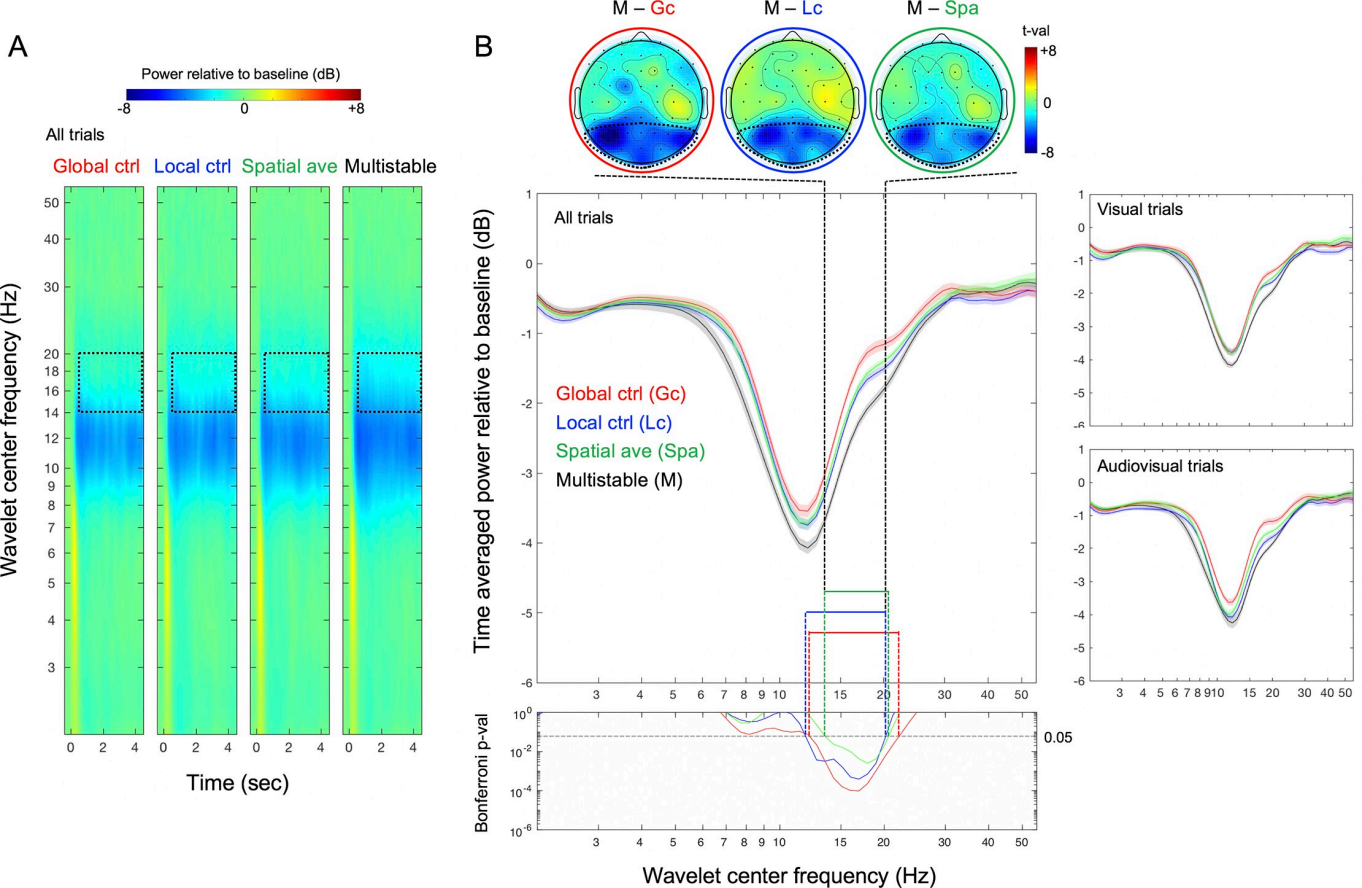
EEG results from Experiment 2. (A) EEG spectral power in dB (baselined to –0.4s to –0.15s prestimulus period) for wavelet center frequencies from 2Hz to 55Hz averaged across posterior sites (indicated with dotted boundaries in the topoplots) as a function of time (relative to flicker onset at 0) for the global-control, local-control, spatial-average, and multistable conditions (from left to right). The dotted rectangles indicate the time period from 0.5s to 4.5s over which EEG spectral power was averaged for analysis and the frequency range from ~14Hz to ~20Hz in which EEG spectral power reductions were significantly larger for the multistable condition than for each of the other conditions (see (B)). (B) Main panel. Time-averaged posterior EEG power (in dB) as a function of wavelet center frequency for the global-control (red), local-control (blue), spatial-average (green), and multistable (black) conditions; the right panels show the data from the visual and audiovisual trials separately. Bottom panel. Bonferroni-corrected *p*-values for the comparison between the multistable condition and each of the other conditions as a function of wavelet center frequency. The tall dashed lines indicate the ~14Hz to ~20Hz frequency range in which EEG power was significantly reduced in the multistable condition relative to all other conditions. The three topographic plots at the top (multistable minus global-control on the left, multistable minus local-control in the middle, and multistable minus spatial-average on the right) show the posterior focus of this multisbability related beta-range power reduction (in *t*-values). The shaded regions represent ±1 *SEM* adjusted for the repeated-measures comparisons (Morey, 2008).
